# Sprouting and Hydrolysis as Biotechnological Tools for Development of Nutraceutical Ingredients from Oat Grain and Hull

**DOI:** 10.3390/foods11182769

**Published:** 2022-09-08

**Authors:** Iván Jesús Jiménez-Pulido, Daniel Rico, Cristina Martinez-Villaluenga, Jara Pérez-Jiménez, Daniel De Luis, Ana Belén Martín-Diana

**Affiliations:** 1 Agrarian Technological Institute of Castilla and Leon (ITACyL), Ctra. Burgos Km 119, Finca Zamadueñas, 47071 Valladolid, Spain; 2 Department of Technological Processes and Biotechnology, Institute of Food Science, Technology and Nutrition (ICTAN-CSIC), Jose Antonio Novais 10, 28040 Madrid, Spain; 3 Department of Metabolism and Nutrition, Institute of Food Science, Technology and Nutrition (ICTAN-CSIC), Jose Antonio Novais 10, 28040 Madrid, Spain; 4 Endocrinology and Nutrition Research Centre, University of Valladolid, Service of Endocrinology and Nutrition, Universitary Clinic Hospital of Valladolid, Av. Ramón y Cajal 3, 47003 Valladolid, Spain

**Keywords:** oat, grain, hull, hydrolysis, antioxidant, glycemic index, anti-inflammatory

## Abstract

Oat consumption has increased during the last decade because of the health benefits associated with its soluble dietary fiber (β-glucan), functional proteins, lipids, and the presence of specific phytochemicals, such as avenanthramides. Oat is consumed mainly as whole grain, and the hull (seed coat), comprising 25–35% of the entire grain, is removed, generating a large amount of waste/by-product from the milling industry. The objective of this study was to evaluate the use of biotechnological strategies, such as sprouting for oat grain (OG) and hydrolysis for oat hull (OH), to enhance antioxidant and anti-inflammatory properties and lower the glycemic index (GI). Sprouting produced significant (*p* ≤ 0.05) increases in free (32.10 to 76.62 mg GAE (100 g)^−1^) and bound phenols (60.45 to 124.36 mg GAE (100 g)^−1^), increasing significantly (*p* ≤ 0.05) the avenanthramide (2c, 2p and 2f) soluble phenolic alkaloid content and anti-inflammatory properties of OG. On the other hand, the hydrolysis of OH using Viscoferm (EH2-OH) and Ultraflo XL (EH21-OH) increased by 4.5 and 5-fold the release of bound phenols, respectively; meanwhile, the use of Viscoferm increased the 4.55-fold soluble β-glucan content in OH, reaching values close to those of OG (4.04 vs. 4.46 g (100 g)^−1^). The study shows the potential of both strategies to enhance the nutritional and bioactive properties of OG and OH and describes these processes as feasible for the industry to obtain an ingredient with high antioxidant and anti-inflammatory activities. Single or combined biotechnological tools can be used on oat grains and hulls to provide nutraceutical ingredients.

## 1. Introduction

Changing people’s lifestyles can result in negative dietary habits, which create nutritional imbalances and increase the prevalence of risk factors for chronic diseases (CD), such as nutrition deficiencies, obesity or hypertension. Consumers are aware of the benefits of a healthy diet. At the same time, the overconsumption of energy-rich foods is pointed out as responsible for the obesity epidemic [1]. Different experts in nutrition have proposed the reduction of cereal intake as an effective solution to reduce the incidence of obesity. However, cereals provide macro (carbohydrates, proteins, fats) and micronutrients (minerals, vitamins), as well as non-nutrient food components, such as dietary fiber, bioactive compounds, and phytochemicals, which are key in the control and reduction of chronic diseases [2]. Indeed, epidemiological studies have provided evidence that the regular consumption of whole cereals significantly reduces the risk of chronic cardiovascular diseases and type 2 diabetes [3,4].

The benefits of cereals vary depending on the form in which they are consumed. The intake of whole grains is increasing, in reference to refined flours, during the last few years; some countries, such as Denmark, have doubled their consumption in the last 10 years [5]. According to the American Association of Cereal Chemists [6], whole grains consist of an intact, ground, cracked or flaked kernel after the removal of inedible parts, such as the hull and husk. Consumption of some cereals in whole form, such as oat and rye, may result in a lower risk of myocardial infarction compared to other cereals [7].

Oat (*Avena sativa*) belongs to the Aveneae tribe of the Poaceae grass family and has been widely cultivated for more than 2000 years. Oat worldwide production ranks sixth, following wheat, maize, rice, barley and sorghum. Over 26 million tons are produced annually and are mainly used for food and livestock feed. The major oat producers are Russia, Canada, Poland, Australia and Finland. Over the centuries, Finns have become specialists in the cultivation and use of oat for its health benefits, even developing gluten-free varieties. Oat hulled varieties are produced in Western countries, while hull-less varieties are more common in China [8].

Oat grain (OG) has an interesting macronutrient composition; it contains unsaturated oleic and linoleic acids, which make up 40% and 36% of the total fatty acid composition, respectively. In addition, oat proteins provide essential amino acids, and OG has a high content in dietary fiber, specially β-glucans (2–8.5%) [9].

Soluble β-glucans (soluble fiber) are key bioactive compounds in oats since they contribute to reduced glycemia and serum cholesterol levels. These health-related properties depend on the viscosity and, therefore, the length of the β-glucan branches, which may be affected (shortened) by harsh processes [10]. The primary mechanism by which β-glucans reduce postprandial glycemia is by increasing viscosity of the chyme, which implies reduction of enzyme-nutrient interactions [11]. On the other hand, the mechanism responsible for the modulation of serum cholesterol by β-glucans is thought to be linked to bile acid metabolism; β-glucans interact with bile acids and prevent their reabsorption in the terminal ileum [10]. Products containing oats are allowed to claim health benefits related to blood cholesterol, according to different regulators [12,13,14,15]. The European Food Safety Authority (EFSA) considers that foods that provide at least 3 g of oat β-glucans per day can claim to produce a lowering effect on blood LDL and total cholesterol.

Other bioactive compounds, besides β-glucans, are present in oats, such as tocopherols, tocotrienols and flavonoids. In addition, it is a unique source of avenanthramides and phenolic amides containing anthranilic acid and hydroxycinnamic acid moieties, compounds that provide antioxidant, anti-inflammatory, and antiproliferative effects [16].

A large quantity of waste/by-product from oat production comes from hull (OH) removal, which makes up 25–35% of the entire grain [17]. Currently, its value is rather low, as it is seen as a waste product. OH composition is mainly crude fiber (lignin, cellulose and hemicellulose) [18]. Hull disposal, because of the large volume produced, can be a serious issue for oat millers. Finely ground hulls can be used for animal feed or as food ingredients. In Northern Europe, some oat mills utilize a hull combustion process to produce energy. OH has previously been of interest to the food industry to help prevent lipid oxidation due to its high antioxidant activity, which comes from its phenolic content [19,20].

In general, phenolic compounds in cereals are integrated through ester and ether linkages in a three-dimensional structure of cellulose, hemicellulose and lignin, resulting in highly bound, low bioavailability characteristics [21,22]. The increased bioavailability of these compounds can be obtained by using cellulolytic and xylanolytic enzymes, which are capable of depolymerizing β-D-(1→4)-glucosidic and β-D-(1→4)-xylosidic bonds [23]. The use of enzymes has been described as an effective strategy to enhance the bioavailability of compounds associated with dietary fiber, increasing their solubilization. Different authors have applied hydrolysis with different enzymes to wheat bran (WB), aiming to increase the content of phenolic compounds [21,23,24]. Enzymatic hydrolysis is a feasible and sustainable strategy with high selectivity when compared to other methods [24].

Another interesting option for enhancing the nutritional and bioactive profile is grain sprouting or germination. This biotechnological tool is considered an effective process for improving the nutritional quality and functionality of whole cereal grains; it is an emerging bioprocess to tailor and improve the nutritional and bioactive properties of grains in a natural way [25,26,27]. Sprouting modifies the nutritional quality of cereals by increasing nutrient digestibility, reducing the level or activity of anti-nutritional compounds, boosting the content of free amino acids and available carbohydrates, and improving the bioactivity of the grain [28,29].

This research evaluated OG sprouting and OH enzymatic hydrolysis as biotechnological strategies for improving the bioaccessibility of antioxidant and anti-inflammatory phytochemicals present in these matrices, with the aim of providing tailored formulations of nutraceutical ingredients with potential health benefits.

## 2. Materials and Methods

### 2.1. Chemicals

Gallic acid (GA), Folin–Ciocalteu (FC) reagent, 6-hydroxy-2,5,7,8-tetramethyl-2-carboxylic acid (Trolox), fluorescein, 2,20-diazobis-(2-aminodinopropane)-dihydrochloride (AAPH), 2,20-azinobis 3-ethylbenzothiazoline-6-sulfonic acid (ABTS^•+^), 2,2-diphenyl-1-picrylhydrazyl (DPPH), iron(III) chloride hexahydrate (FeCl_3_∙6H_2_O), 2,4,6-tripyridyl-triazine (TPTZ), iron(II) sulfate heptahydrate (FeSO_4_∙7H_2_O), gallic acid, apigenin, ferulic acid, avenanthramide C, *p*-coumaric acid and sinapic acid were obtained from Sigma-Aldrich, Co. (St. Louis, MO, USA). Amyloglucosidase (EC 3.2.1.3) and glucose oxidase-peroxidase (GOPOD) were provided by Megazyme (Wicklow, Ireland). Glacial acetic acid, sodium acetate and chlorhydric acid were obtained from PanReac AppliChem (ITW Reagents, Darmstadt, Germany). The solvents were HPLC-grade (Sigma Aldrich Co., Madrid, Spain, and Merck KGaA, Darmstadt, Germany). Food-grade enzymes UltraFlo XL and Viscoferm were kindly provided by Novozymes (Bagsværd, Copenhagen, Denmark).

### 2.2. Materials

Oat (*Avena sativa* L., var. Chimene) dehulled grain and hull were kindly provided by Sdad. Coop. Regional Ltd.a. Ribera del Duero (Burgos, Spain). Chimene is a white grain variety cultivated in winter with a high protein content and productivity. Samples were grown in Burgos (Spain) during the campaign 2019–2020, and oat was dehulled in the provider facilities using a mechanic system in dry conditions to separate the grain from the hull. The samples were transported to ITACyL and milled before being stored in plastic bags under vacuum conditions until further analyses.

### 2.3. Biotechnological Strategies to Enhance Nutraceutical Properties

#### 2.3.1. Sprouting

OG were germinated following the method described by Tomé-Sánchez et al. [27] with some modifications. First, OG were visually inspected to ensure there was no contamination of other grains and afterwards sanitized using tap water with 0.5% sodium hypochlorite(*v*/*v*) in a ratio 1:6, *w*/*v* for 30 min. Grains were rinsed with tap water to neutralize the pH and soaked in distilled water (1:6 ratio, *w*/*v*) for 4 h at room temperature. After that time, the soaked OG were spread on wet filter paper on a plastic rack and placed in plastic trays with tap water. Grains were covered with moist filter paper and placed in a germination cabinet (Snijders Scientific, Tilburg, The Netherlands), which provided a relative humidity >90%. Sprouting was carried out using conditions optimized in darkness at 21 °C for 5 days. Sprouted oats (SO) were submitted to a high-pressure process (HPP) (Wave 6000/135, Hiperbaric, Burgos, Spain) at 6000 MPa for 5 min and freeze-dried (LyoQuest, Telstar, Barcelona, Spain). Subsequently, the sprouts were milled to a particle size of 0.5 mm. The flours were stored in vacuum plastic bags until analysis.

#### 2.3.2. Enzymatic Hydrolysis

Enzymatic hydrolysis was performed following the method of Martín-Diana et al. [30], with some modifications. OH was resuspended in water (1:20 *w*/*v*). The solution was submitted at high hydrostatic pressure (HHP, 6 × 108 Pa, 5 min) using an HHP unit (Wave 6000/135, NC Hyperbaric, Burgos, Spain) with a vessel of 135 L and 200 mm diameter. After the batch was treated using a hydrothermal machine performed at 121 °C, 1.2 × 10^5^ Pa for 15 min using Ilpra Plus 100 autoclave equipment (Ilpra Systems, Barcelona, Spain). Subsequently, 1.5 M malic acid was used to adjust the pH to 5 prior to enzyme incorporation. Immediately thereafter, one part of batch was incubated with Novozymes food grade UltraFlo XL and in the other batch with Viscoferm, both at 1% (enzyme to OH dry weight ratio, w:w), and enzymatic hydrolysis was performed at 47 °C for 20 h using a temperature-controlled water bath with magnetic stirring at 1000 rpm (Unitronic Vaivén C, Selecta S. A., Spain), resulting in enzymatic hydrolysates EH1-OH and EH2-OH, with UltraFlo XL and Viscoferm, respectively. At the end of the incubation period, enzymes were inactivated in a water bath at 95 °C for 10 min. Insoluble residues were removed by filtration using a nylon filter (200 μm-mesh). Finally, OH soluble fractions were filtrated and stored at 4 °C and immediately analyzed.

### 2.4. Nutritional Characterization

The moisture content was determined by drying the powdered sample (OG, OH and SO) at 105 °C for 3 h. For total fat content, a Soxtec extracting unit (AOAC 2005, method 2003.05) [31] was used with petroleum ether extraction (40–60 °C) for 4 h. Dumas method 990.03 [31] was performed for total protein content in an elemental analyzer (LECO Corp., St. Joseph, MI, USA). A conversion factor of 6.25 was used to convert nitrogen into protein values. To determine the ash content, the samples were incinerated in a muffle furnace at 550 °C for 5 h (AOAC 2005, method 923.03) [31]. Carbohydrates were estimated by difference.

Total dietary fiber (TDF) content was evaluated using the TDF100A-1KT assay kit provided by Sigma (St. Louis, MO, USA), based on the AOAC method 985.29 [31].

β-glucan content was quantified by a 1.3:1.4 mixed-linkage β-glucan kit (Megazyme, Ireland), following the manufacturer’s instructions. The assay uses lichenase and β-glucosidase to hydrolyze β-glucan to glucose. Subsequently, glucose reacts with GOPOD (glucoseoxidase/peroxidase) reagent, and the absorbance was measured at 510 nm in a microplate reader (Spectrostar Omega, BMG Ortenberg, Germany). All measurements were performed in duplicate. Results were expressed as g β-glucan (100 g)^−1^ (d.m.).

Total starch content (TSC) and phytic acid/total phosphorus (PA) were determined using K-TSTA-100A and K-PHYT assay kits (Megazyme, Wicklow, Ireland), respectively. The results were corrected for moisture content and expressed as g (100 g)^−1^ of dry matter (d.m.). All analyses were performed in duplicate.

The fatty acid profile was determined for all grains and bran flours. Lipids were extracted according to the method of Bligh and Dyer [32]. The lipid-containing chloroform phase was separated and evaporated. The remaining phase was dissolved in 1 mL of hexane, and a methylated procedure was carried out by adding 100 µL of 0.5 M methanolic KOH and leaving the reaction for 10 min at room temperature. The upper layer was transferred to a 2 mL vial. The analysis of fatty acid methyl esters (FAME) was carried out on a gas chromatograph Agilent 7890A (Agilent Technologies, Santa Clara, CA, USA) with a flame ionization detector. A DB-23 column 60 m × 0.32 mm, (0.25 m film thickness). Helium was used as the carrier gas. The oven temperature was programmed to 50 °C for the first 7 min and increased up to 200 °C at a rate of 25 °C per min; then, the temperature was further increased to 230 °C at a rate of 3 °C per min and held for 26 min. The injection and detector temperatures were 250 °C and 280 °C, respectively. One microliter of the hexane extract was injected in split mode (ratio 25:1), and FAME’s were identified by comparison of retention times with those of the standard (37 FAME’s mix, Supelco, Sigma-Aldrich). Results were expressed as a percentage of total fatty acids.

### 2.5. Phenolic Extract Preparation

Free and bound phenolic compounds were extracted, following the procedure described by Dinelli et al. [33], from different samples: oat hull (OH), oat grain (OG) and sprouted oat (SO).

#### 2.5.1. Release of Free Phenolic Compounds (FP)

One gram of each sample was extracted with 20 mL of chilled EtOH/H_2_O (80:20, v/v) by magnetic agitation for 10 min at room temperature (RT). Supernatant was collected after centrifugation (25 °C, 2500 × *g*, 10 min), and the extraction was repeated twice. Both supernatants were pooled, evaporated on a rotary evaporator (Rotavapor R-210, Buchi, Switzerland) at 45 °C under vacuum and, finally, dried under continuous and gentle flow of nitrogen gas. The extracts were reconstituted in 10 mL of MeOH/H_2_O (80:20, v/v), filtered through a nylon filter (0.22 μm, 25 mm) and stored at −80 °C until analysis.

#### 2.5.2. Release of Bound Phenolic Compounds (BP)

The pellet obtained after centrifugation during the extraction of free phenolic compounds (2.5.1.) was subjected to alkaline and acid hydrolysis to recover the bound phenolic compounds. A total of 12 mL of distilled water and 5 mL of 10 M NaOH were added to the residue and stirred overnight at room temperature using a magnetic stirrer. The pH of the solution was adjusted to pH 2, and the released phenolic compounds were extracted three times with 15 mL of ethyl acetate by manual shaking and centrifugation (25 °C, 2500× *g*, 10 min). The ethyl acetate layers were polled and refrigerated.

After alkaline hydrolysis, acid hydrolysis was carried out by adding 2.5 mL of concentrated HCl and incubated in a water bath at 85 °C for 30 min. The sample was cooled down and phenolic compounds were extracted with ethyl acetate in the same way as described above. Fractions obtained from alkaline and acid hydrolysis were mixed and evaporated to dryness with a rotary evaporator (40 °C). The extracts were reconstituted with 10 mL of MeOH, filtered through a nylon filter (0.22 μm) and stored at −80 °C until analysis.

### 2.6. Determination of Total Phenolics (TPs)

TPs were measured according to Slinkard and Singleton [34] with the Folin–Ciocalteu phenol reagent in the free and bound phenolic compound fractions. Absorbance was measured at 765 nm using a microplate reader (Fluostar Omega, BMG, Ortenberg, Germany). Gallic acid was used as the standard (700–98 μM). The results were expressed as mg gallic acid equivalents (GAE) (100 g)^−1^ d.m. All analyses were performed in duplicate.

### 2.7. Characterization of Phenolic Compounds by HPLC-ESI-QTOF-MS

Free and bound polyphenol fractions of OG, OH and SO were injected directly. For separation, HPLC (Agilent 1200, Agilent Technologies, Santa Clara, CA) with DAD (Agilent G1315B) and a QTOF mass analyzer (Agilent G6530A) with atmospheric pressure electrospray ionization (ESI) were used. The column used was 250 mm × 2 mm i.d., 5 μm, Luna C_18_ (Phenomenex, Torrance, CA) at 25 °C. For gradient elution, 0.1% aqueous formic acid (solvent A) and 0.1% formic acid in acetonitrile (solvent B) were used. The following gradient was applied at a flow rate of 0.4 mL/min: 0 min, 8% B; 10 min, 23% B; 15 min, 50% B; 20 min, 50% B; 23 min, 100% B, followed by a re-equilibration step. The injection volume was 2 μL. Negative ion mode with a mass range of 100–1200 Da, a source temperature of 325 °C and a gas flow of 10 L h^−1^ were applied for data acquisition. Peak identity was compared with the retention times of commercial standards when available. In addition, the molecular formula proposed by the MassHunter Workstation software version 4.0 for the different signals obtained in the MS experiments was compared with previously reported phenolic compounds in oat and other cereals, and a maximum error of 10 ppm was accepted. For MS/MS experiments, the auto MS/MS acquisition mode was used; the main fragments were compared with the fragmentation patterns reported for phenolic compounds.

Phenolic compounds were quantified with calibration curves of authentic standards (gallic acid, apigenin, ferulic acid, avenanthramide C, (-)-epicatechin, secoisolariciresinol, kaempferol, sinapic acid and *p*-coumaric acid) at a concentration range between 0.1 and 25 μg mL^−1^, showing good linearity (R^2^ > 0.99). The results were expressed as the mean and standard deviation of two independent replicates in mg (100 g)^−1^ sample (d.m.).

### 2.8. Total Antioxidant Capacity (TAC)

TAC was determined in the free and bound phenolic compound fractions by ABTS^•^+ radical cation scavenging activity, oxygen radical absorbance capacity (ORAC) and ferric reducing antioxidant power (FRAP) assays. All analyses were performed in duplicate.

#### 2.8.1. ABTS^•+^ Radical Cation Scavenging Activity (ABTS^•+^)

ABTS^•+^ assay, based on Re et al. [35], was carried out as modified by Martin-Diana et al. [36]. In a 96-well microplate, 20 μL of sample was mixed with 200 μL of ABTS^•+^ working solution. After 60 min, absorbance was measured at 734 nm with a microplate reader (Spectrostar Omega, BMG Ortenberg, Germany). A Trolox curve was prepared as a standard (7.5–210 μM). The results were expressed as μmol TE (100 g)^−1^ sample (d.m.).

#### 2.8.2. Oxygen Radical Absorbance Capacity (ORAC)

The ORAC assay was carried out according to the method reported by Ou et al. [37], with modifications. Phosphate buffer (10 mM, pH 7.4) was used to dilute the samples and the Trolox standard curve (7.5–210 μM). In a black 96-well microplate, a volume of 25 μL of Trolox standard, sample and phosphate buffer as blank and a volume of 125 μL of fluorescein were added. They were incubated at 37 °C for 3 min before adding 25 μL of AAPH solution to initiate the oxidation reaction. Fluorescence was monitored for 120 min with a microplate reader (Fluostar Omega, BMG, Ortenberg, Germany) using 485 nm excitation and 520 nm emission filters. Results were calculated by plotting the areas under the fluorescein decay curves between blank and sample and expressed as μmol TE (100 g)^−1^ sample (d.m.).

#### 2.8.3. Ferric Reducing Antioxidant Power (FRAP)

FRAP was determined following the procedure reported by Benzie and Strain [38], with some modifications [39]. A 300 mM acetate buffer pH 3.6, a 10 mM TPTZ (2,4,6-tripyridyl-triazine) solution in 40 mM HCl, and a 20 mM FeCl_3_∙6H_2_O solution were prepared. FRAP working solution was prepared by mixing the acetate buffer, TPTZ solution and FeCl_3_∙6H_2_O solution in a 10:1:1 ratio of volumes. A curve of FeSO_4_∙7H_2_O was prepared as standard (400–3000 μM). 20 μL of sample, standard or distilled water as blank was mixed with 1.9 mL of FRAP working solution in Eppendorf tubes. They were stirred and incubated for 5 min. Absorbance was measured at 593 nm in a 96-well plate in a microplate reader (Spectrostar Omega, BMG Ortenberg, Germany). The results were expressed as mmol of Fe Equivalents (FeE) (100 g)^−1^ sample (d.m.).

### 2.9. Glycemic Index (GI)

GI was determined following the method described by Gularte and Rosell [40], with slight modifications. Samples containing 50 mg of available starch were dissolved in 2 mL of Tris-maleate buffer (0.1 M, pH 6) and then 2 mL of enzyme solution containing porcine pancreatic amylase (460 U mL^−1^) and amyloglucosidase (6.6 U mL^−1^) were added. Aliquots of 150 μL were taken at different times during the incubation period (0, 10, 20, 20, 30, 60, 60, 90 and 120 min) and the enzymatic reaction was immediately stopped in boiling water for 5 min and cooled on ice. Following this, a volume of 150 μL of absolute ethanol was added and the sample was centrifuged (10,000× *g*, 5 min). The pellet was washed with 200 μL of EtOH:H_2_O (1:1, *v*/*v*). The sample was stirred and centrifuged (10,000× *g*, 5 min), and the supernatants were pooled. Subsequently, a GOPOD kit (Megazyme, Bray, Ireland) was used to perform the colorimetric analysis of glucose. The values of the hydrolysis index (HI) and glycemic index (GI) were calculated using the formula proposed by Granfeldt [41].

### 2.10. Determination of Anti-Inflammatory Activity (AIA)

The cell viability of murine RAW 264.7 macrophages (American Type Culture Collection, Manassas, VA, USA) was determined to address the cytotoxicity of the phenolic extracts. Stock solutions (10 mg/mL) of phenolic extracts were prepared in dimethyl sulfoxide and sterile filtered with a 0.22 μm polyvinylidene fluoride. Cells were grown in Dulbecco’s Modified Eagle Medium (DMEM, Life Technologies, Carlsbad, CA, USA) containing 10% (*v*/*v*) heat-inactivated fetal bovine serum (FBS, Life Technologies, Carlsbad, CA, USA) and 1% penicillin/streptomycin (Life Technologies, Carlsbad, CA, USA) at 37 °C with 5% CO_2_. Cell viability was determined using an MTS assay [27]. Briefly, cells were seeded in 96-well plates at a density of 5 × 10^4^ cells/well. After overnight attachment, the cells were treated with 0.5 mg/mL of phenolic extracts diluted in growth medium with the presence of 0.1 μg/mL of lipopolysaccharide from *Escherichia coli* O55:B5 (Sigma-Aldrich, St. Louis, MO, USA) for 24 h. After incubation, the cell culture media were collected for cytokine quantification and cells were treated with the Cell Titer 96 Aqueous One Solution Cell Proliferation Assay (Promega, Madison, WI, USA).

Cytokine analysis of the cell culture medium of macrophages was performed using the Mouse Cytokine Magnetic kit (Milliplex MCYTOMAG-70K-06, Merck Life Sciences, Madrid, Spain). This cytokine panel allows the simultaneous quantification of 5 mouse cytokines/chemokines, including MCP-1, IL-1β, IL-6, IL-10, INF-γ and TNF-α, based on fluorescence-encoded beads suitable for flow cytometry. A multiplex immunoassay was performed following the manufacturer’s recommendations. Data were acquired on a Luminex XYP flow cytometer (Luminex Co., Austin, TX, USA) and analyzed using the Belysa^TM^ Data Analysis Software (version 1.2). MCP-1 was over the detection limit, whereas INF-γ was below the lower threshold in all the analyzed samples; thus, they were excluded from the analysis.

### 2.11. Statistical Analysis

Analysis of variance (ANOVA) and Duncan’s post hoc test were performed to detect differences between the mean values. All statistical analyses, except quantification of phenolic compounds by HPLC-ESI-QTOF-MS, were performed with Statgraphics Centurion XVI^®^ (StatPoint Technologies, Inc., Warrenton, VA, USA). The results were expressed as mean ± standard deviation. Principal component analysis (PCA) was performed on standardized data to elucidate the relationships among the variables.

The phenolic compound data quantified by HPLC-ESI-QTOF-MS were analyzed using IBM SPSS Statistics 28.0. Normality of the data was tested using the Shapiro–Wilk test. Due to the absence of normality, the Kruskal–Wallis test and Mann–Whitney’s U test were performed for comparisons between unrelated groups. The results were expressed as mean values with their standard deviations. Significance was defined as a *p*-value < 0.05.

## 3. Results

### 3.1. Nutritional Characterization

Oat dehulled grain (OG), hull (OH) and sprouted grain (SO) were characterized in their nutritional composition (protein, ash, fat, carbohydrates, total dietary fiber), fatty acid composition, and phytic acid (PA) content, in order to better understand differences in bioactivity that may be associated with nutrient and antinutrient content (Table 1).

The results showed that ash content in OG and SO ranged between 2.41 and 2.50 g (100 g)^−1^, without significant (*p* ≥ 0.05) differences between them; on the other hand, OH ash content was almost double (4.3 g (100 g)^−1^); this is expected, since most minerals are located in the outer layers [42]. Similar ash levels were observed in OG and SO, so sprouting did not affect mineral content. OG ash values were significantly (*p* ≤ 0.05) higher than the results reported by the authors in previous studies [43], although the values were in the range with other studies (2.7–3.5 g (100 g)^−1^) [44,45,46]. However, OH ash values were lower than those reported in previous studies, ranging from 5.2 to 6.3 g (100 g)^−1^ [44,45,46]. Probably, these observed differences might be associated with genetic variety, dehulling or agronomic practices. According to the bibliography, potassium is by far the most abundant mineral, followed by calcium, magnesium, phosphorus and sulfur [17].

As it occurs to other cereals, total carbohydrates account for the largest macronutrient fraction in oat. Carbohydrates were evaluated in all the samples (Table 1); OG showed values close to 79.7 g (100 g)^−1^, similar to previously reported values in oat [44] and to the carbohydrate content of other cereals, such as barley, maize, wheat or rice (78.8 g (100 g)^−1^, 76 g (100 g)^−1^, 76.6 g (100 g)^−1^ and 81.5 g (100 g)^−1^) [47]. After sprouting, a significant (*p* ≤ 0.05) reduction in carbohydrate content was observed, which can be associated with increasing α-amylase activity, an enzyme that hydrolyses α-1,4 glucosidic linkages of starch [48]. The content of carbohydrates in the seed coat (OH) was significantly (*p* ≤ 0.05) higher than in the OG and OS, with values close to 90 g (100 g)^−1^. Since carbohydrates include total dietary fiber (TDF) and starch (TS), both fractions were also evaluated.

TDF determination can also be relevant for antioxidant activity, since TDF covalently links to polyphenols with bioactive properties, such as antioxidant activity, anti-inflammatory activity or low glycemic index. The highest contribution of phenolics in wholegrain and seed coat is in the form of insoluble compounds, which are bound through ester and ether linkages to polysaccharides (arabinoxylan and lignin) of the cell wall [43,49,50].

TDF was evaluated considering the Codex Alimentarius dietary fiber definition: “carbohydrate polymers with 10 or more monomeric units” [51] in all the samples (OG, OH and SO). The results (Table 1) showed that TDF values in native grain (OG) were higher than values reported previously by the authors (12.63 g (100 g)^−1^ vs. 11.72 g (100 g)^−1^) [43] and Šterna et al. [52] (10.3 g (100 g)^−1^), although similar to values reported by Dhingra et al. [53] for oat varieties. These differences may be associated with genetic variation and agronomic practices, among other reasons. After sprouting, a significant (*p* ≤ 0.05) reduction in TDF values (12.63 g (100 g)^−1^ in OG vs. 8.81 g (100 g)^−1^ in SO) was observed; this behavior was associated with cell wall material degradation after sprouting [54]. Ghavidel et al. [55] reported an increase in soluble and total dietary fiber fractions but a decrease in insoluble dietary fiber due to the effect of α-amylase.

The TDF content in oat hulls (OH) was significantly (*p* ≤ 0.05) higher than that found in OG and SO, reaching values of 89.64 g (100 g)^−1^, values in agreement with other studies [56], with a lignocellulosic composition [57]. The largest fraction of the hull corresponds to hemicellulose, followed by lignin, which is the majority of the lignin as acid insoluble, and finally cellulose; this is in contrast to other agricultural waste products, which are typically richer in cellulose and poorer in lignin [58].

The water soluble β-glucan was also determined (Table 1) due to the important concentration of this type of bioactive soluble fiber in oat grain, and likely affected by sprouting and hydrolysis processes. The OG β-glucan content was 4.46 g (100 g)^−1^, slightly higher than the values reported by the authors in a previous work (3.06 g (100 g)^−1^) [43], although similar to the results described by Saastamoinen et al., who reported β-glucan values from 3.0 to 4.7 g (100 g)^−1^ in hulled oat cultivars grown in Finland [59,60]. SO samples showed a significant (*p* ≤ 0.05) decrease of β-glucan from 4.46 g (100 g)^−1^ to 1.55 g (100 g)^−1^, which can be associated with an increment in enzymatic activity as part of the sprouting process, increasing β-glucanase activity; this increase was observed from day 3 to 6 of sprouting, and it was favored by temperatures higher than 15 °C [61]. A similar behavior was described by Aparicio-García et al. [25], who found a β-glucan 40% decrease, when compared with sprouted and non-sprouted grains. The OH had values lower than 0.2 g (100 g)^−1^ as expected, in agreement with Dziki et al. [56], who reported the seed coat as very poor in soluble fiber (β-glucan), as compared to grain. However, after enzymatic treatment of OH, the content in β-glucan increased significantly (*p* < 0.05), by 7.75-fold-times with UltraFloXL (EH1-OH) and 22.7-fold-times (4.04 mg (100 g)^−1^) with Viscoferm (EH2-OH), as can be observed in Supplementary Figure S1. These results agree with previous studies that reported the hydrolysis of cellulose, hemicellulose, and β-glucans [62].

Starch was also measured (Table 1). The total starch content (TSC) was evaluated in the oat grains before and after sprouting. OG and SO showed similar levels of starch, with a small increase in the case of SO. The TSC content in OH was residual. Oat hulled varieties have been reported to contain lower α-amylase activity; it has also been reported that higher humidity in the grain during sprouting, favored by the presence of the hull, can result in an inhibitory effect on α-amylase enzymatic activity [63]. This lack of starch degradation observed during sprouting is an important aspect with relevance for healthier product development, as it results in a lower glycemic index.

Oat macronutrient composition is unique among cereals due to its relatively high oil content [59,64]. In addition, lipids are located throughout the kernel, while lipids in most other cereals are concentrated in the embryo [65]. Crude fat (Table 1) values differed significantly (*p* < 0.05) among studied samples; OG had values of 6–7 g (100 g)^−1^, being values similar to other studies previously reported by the authors [43], and in the range of values described by other groups, who reported between 5 and 9 g (100 g)^−1^ [44,66]. SO showed a 20% increase in fat content after sprouting, in agreement with other studies that found a similar trend [64]. Outinen [67] studied the influence of sprouting conditions on the lipid content and reported no lipid degradation or significant formation of free fatty acids during sprouting, also describing that the lipolytic activity of hulled oat remained stable or decreased during sprouting. Other authors have described that this effect was more evident in dehulled oat, and similar findings were reporter later by Aparicio-García et al. [68], when compared lipase activity in hulled and dehulled sprouted oat, suggesting that there must be an important lipase activity in oat hull since the lack of hull in oat during sprouting reduce significantly lipase activity and the reduction in crude fat.

On the other hand, OH shower a poor fat content, the values were very low compared to OG and OS, the fat content was lower of 1% (0.63 g (100 g)^−1^), the low lipid content, which is in line with previous findings reported by other authors such as Bryngelsson et al. [69] who reported values between 0.5 and 1.4 g (100 g)^−1^.

The fatty acid profile was evaluated (Table 1); unsaturated fatty comprised more than 82% of the total fatty acid composition, with linoleic (C:18:2 (n6)), oleic (C:18:1(n9)), and palmitic (C:16:0) being the main fatty acids found. The results were similar to those reported in the bibliography by different groups [43,70]. The sprouting increased significantly (*p* ≤ 0.05) the SFA and MUFA content. The analysis of specific compounds showed that sprouting increased oleic acid by 15%, while linoleic acid decreased by 2.6%. Stearic acid (C18:0) was detected only after sprouting, although at a low concentration (2 g (100 g)^−1^). The importance of stearic acid and its health-related effects (reduced blood pressure, improved heart function, and reduced cancer risk) compared to other saturated fatty acids (e.g., palmitic acid) has been reported [71,72]. The hull showed a very different fatty acid profile from that of the whole and sprouted grains; a higher ratio of SFA with respect to unsaturated fatty acid (MUFA + PUFA) was observed. OH had the lowest concentration of linoleic acid (C:18:2 (n6)) and the absence of C18:0.

Protein was analyzed (Table 1) in oat samples due to its importance from nutritional and bioactive points of view. The results showed that the native grain (OG) had protein values similar to those reported in other studies [43]; in contrast, the sprouted grain showed lower protein values than the native grain, probably due to the action of proteinases from the aleurone layer into the endosperm of the grain, thus favoring the degradation of proteins in the endosperm into transportable peptides. González-Montoya et al. [73] demonstrated proteolysis during sprouting, which enhanced the release of bioactive peptides.

Antinutrients were evaluated since they play an important nutritional detrimental role, as in the case of the absorption of certain types of minerals, such as iron [74]. Phytic acid (PA) was measured because it is present at high concentrations in cereals. OG showed values similar to those reported in oat by other authors (0.94 g (100 g)^−1^), the sprouting as it has been described by different authors reduced the content of this compound (12% respect to native oat grain). Oat phytate is difficult to degrade [75] and degradation of phytate during sprouting is lower in oat than in other cereals [76]. OH phytate content was also evaluated; the hull showed a very low concentration of PA, 0.1 g (100 g)^−1^, which might represent an important advantage for its use as an ingredient. After hydrolysis (Supplementary Figure S2), an increment of up to 0.42 g (100 g)^−1^ in PA was observed when hydrolysis was carried out with UltrafloXL, and to 0.30 g (100 g)^−1^ when it was done with Viscoferm, levels were significantly lower than those found in the grains.

### 3.2. Determination and Characterization of Total Phenolic Compounds (TPC)

Total phenolic content (TPs) was evaluated in OG, OH and SO samples after two different extraction procedures, with the aim of extracting free (FP) and bound (BP) compounds. TP was also evaluated in the OH-hydrolyzed samples (EH1-OH and EH2-OH). The OG TP values were significantly (*p* ≤ 0.05) higher in BP with respect to FP (32.10 vs. 60.45 mg GAE (100 g)^−1^), and both fractions were lower than the values reported by Aparicio-Garcia et al. [68], which found a significantly higher, near double, TP content in free and bound fractions (221–227 (100 g)^−1^). However, the results of the study were similar to those obtained by other authors [77], who reported an average TP value close to 99 mg GAE (100 g)^−1^. Differences found in the literature can be due to many factors, such as the presence or lack of hull in the varieties studied, environmental conditions or agronomic practices, among others [68,77].

**Figure 1:**
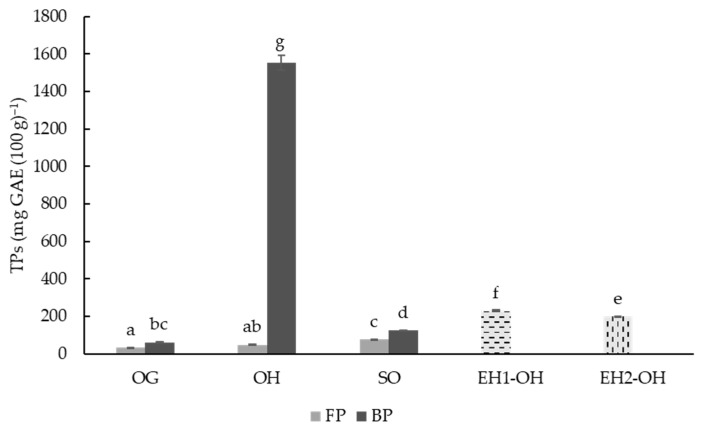
Total phenolic (TP) content (mg GAE (100 g)^−1^) for free polyphenolic fraction (FP) and bound polyphenolic fraction (BP) of oat grain (OG), oat hulls (OH), sprouted oat (SO), enzymatic hydrolysate oat hulls with UltraFloXL (EH1-OH) and enzymatic hydrolysate oat hulls with Viscoferm (EH2-OH) samples. The results were expressed as mg GAE (100 g)^−1^ of dry matter. Different letters indicate significant differences (*p* < 0.05).

The sprouting produced a significant (*p* ≤ 0.05) increase in TPs in the free (from 32.10 to 76.62 mg GAE (100 g)^−1^) and bound fractions (from 60.45 to 124.36 mg GAE (100 g)^−1^), as compared to the native non-germinated grain (OG). This increase has been reported in different cereals, including oat grains [78]. The results agreed with the study reported by Oksman-Caldentey et al. [79], who also observed a TP increase in oats during sprouting. This was partially explained by better phenolic compound extractability from kernel structures; these authors also reported that, after sprouting, most of the phenolic compounds were bound in ester form or in glucosidic forms, which was in line with the findings reported in this study, where the content of bound phenolics was twice that of free phenols (Figure 1). Moreover, it has been identified that this increment can be due to *de novo* synthesis during sprouting in the embryonic axis of sprouted grain [80]. However, it is important to highlight that sprouting conditions (time and temperature) have been identified as key factors determining this increment in phenolics, and under certain conditions, sprouting times of 156–216 h may be needed to reach significant increments [68]. The OH- and OG-free phenolic fractions were not significantly different, but the OH-bound fraction was between 12 and 25 times that of SO and OG, respectively. These results were expected since the hull is rich in non-soluble phenolic compounds linked to the cell wall. Previous studies by Piatkowska et al. [81] on husk, bran, endosperm and whole grain of 13 oat varieties concluded that OH contained the highest level of polyphenols [81].

UltraFloXL (EH1-OH) and Viscoferm (EH2-OH) were also evaluated due to their cellulosic and hemicellulosic activities and the efficiency of releasing and increasing the bioavailability of phenolic compounds in WB [20]. Bautista-Expósito et al. reported that the solubilization yield of TPs from wheat bran depended on the type of enzyme(s) and reaction conditions selected during enzymatic treatments (enzyme to substrate ratio, pH, temperature, time, etc.) [20]. UltraFloXL and Viscoferm were selected because they produced better yields among other enzymes evaluated by the authors in terms of solubilization of bound phenols in cereal brans [20]. The results showed (Figure 1) that the use of enzymes increased soluble phenolics by 4.5–5 times. Although the efficiency in terms of phenolic release was higher with UltraFloXL than with Viscoferm, both enzymes increased the available phenolic compounds when compared to non-hydrolyzed OH (49.87 mg GAE (100 g)^−1^) or SO (124.36 mg GAE (100 g)^−1^).

**Figure 2:**
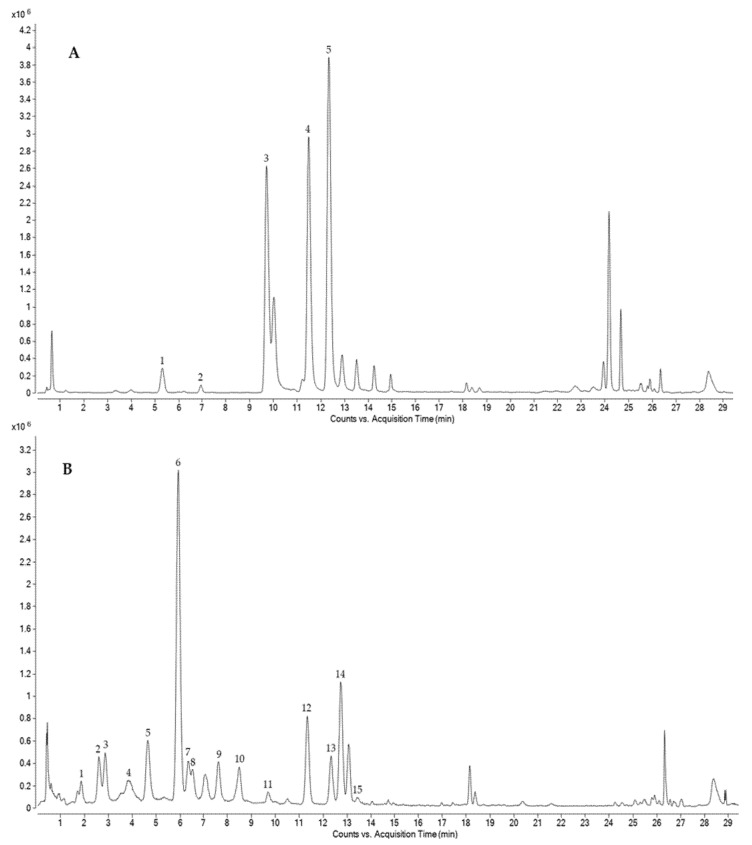
(**A**) Combined extracted ion chromatograms for identified phenolic compounds in the free fraction of oat: 1 = 1-O-sinapoyl-beta-D-glucosa, 2 = apigenin-6-Carabinoside-8-C-hexoside III, 3 = avenanthramide C, 4 = avenanthramide 2p, 5 = avenanthramide 2f. (**B**) Combined extracted ion chromatograms for identified phenolic compounds in the bound fraction of oat: 1 = hydroxybenzoic acid, 2 = caffeic acid, 3 = 4-hydroxybenzaldehyde, 4 = protocatechuic acid, 5 = p-coumaric acid, 6 = ferulic acid, 7 = sinapic acid, 8 = isoferulic acid, 9 = diferulic isomer 1, 10 = diferulic isomer 2, 11 = avenanthramide C, 12 = diferulic isomer 4, 13 = avenanthramide 2f, 14 = diferulic isomer 5, 15 = diferulic isomer 6.

Eighteen phenolic compounds were identified (Table 2) in the different oat samples and classified as hydroxybenzoic acids (2), hydroxycinnamic acids (14), flavones (1) and hydroxybenzaldehyde acids (1) after HPLC-ESI-QTOF-MS analyses. Figure 2 shows the chromatograms of the phenolic profiles of free and bound fractions. The previously described characteristic fragments of the phenolic compounds were used to confirm their identification. The loss of methyl radicals (15 units) by collision-induced dissociation was observed in several compounds, such as sinapic and ferulic acids. Additionally, a loss of carbon dioxide ion (44 units) was detected in *p*-coumaric acid, ferulic acid and avenanthramides. This fragmentation pattern has been previously observed in the analysis of phenolic acids [82] and avenanthramides identified in oat grains [83]. In addition, in some diferulic isomers, fragments corresponding to the monomers (ferulic acid with *m*/*z* 193) were detected.

This also happened with 1-O-sinapoyl-beta-D-glucose, where one of its fragments, *m*/*z* 223, corresponded to sinapic acid. Identified compounds were confirmed by the low errors obtained in the samples, less than ±10 ppm (Table S1 shows the experimental and calculated m/z values for each compound and sample). A higher number of compounds was found in the OG and SO samples than in the OH samples. Additionally, EH1-OH and EH2-OH presented 4 and 5 identified compounds, respectively.

This seems to indicate that enzymatic treatment reduced the diversity of phenolic compounds present on the oat hull. Although enzymatic hydrolysis has previously shown potential for the release of phenolic fractions in cereal by-products [22,24,33], some authors have found otherwise, and a reduction in phenolic content after enzymatic hydrolysis was observed [84,85]. These later results have been explained by the degradation of phenolic compounds due to excess enzymatic activity.

Regarding the distribution of phenolic compounds among free and bound fractions, those identified were found mainly in the bound fraction, particularly for SO. Avenanthramides are soluble phenolic acids characteristic of oats of high interest due to their high antioxidant capacity, which can be in the order of 10 to 30 times that of some other phenolic compounds found in this cereal [86]. The levels of avenanthramides identified were much higher in SO (sum of avenanthramide 2c, 2p and 2f: approx. 50 mg (100 g)^−1^) than in OH (approx. 4 mg (100 g)^−1^) and were identified to a very low concentration in OG (avenanthramide 2f: 0.23 mg (100 g)^−1^). Previously, sprouting has been shown to favor the presence of these compounds in oats [87].

Table 3 shows the phenolic compound content in the different oat samples. Overall, the total phenolic compound content ranged from 23.08 to 386.71 mg (100 g)^−1^, corresponding to the EH2-OH and OG samples, respectively. These values are different from the values previously obtained by our group [43], which may be due to varietal diversity, growing stage and processing methods. The free polyphenol content ranged between 2.1% and 49.4% of the total phenolic compounds in OG and SO, respectively; in this fraction, the most abundant compound in each sample was different. Conversely, bound polyphenols, i.e., phenolic compounds linked to dietary fiber, ranged from 58.8% to 97.9%. In contrast to the diversity observed in free polyphenols, the main compound in bound polyphenols was ferulic acid in all cases, reaching 213.76 mg (100 g)^−1^ in OH. These results agree with those obtained in other studies. For instance, Martin-Diana et al. [43] observed ferulic acid and its dimers as the main constituent in wheat and oat (hull and grain) bound polyphenol fractions. Mathew and Abraham [88] reported that ferulic acid is covalently linked to polysaccharides and components of lignin, being found in a higher proportion as bound polyphenols.

Concerning the differences between samples, the amount of total phenolic compounds, expressed as the sum of the free and bound polyphenols, was higher in OH than in OG (386.71 ± 4.85 mg (100 g)^−1^ vs 65.79 ± 1.97 mg (100 g)^−1^), although, as stated, the diversity of compounds in OH was lower. In this sense, Calinoiu and Vodnar [89] also observed that phenolic compounds are found in higher amounts in the hull than in the whole grain after studying different varieties of cereals. Similarly, Vitaglione et al. [90] reported that cereal variety and its processing (milling) conditions are factors that can affect the amount of phenolic compounds, being different between the hull and the whole grain. However, the total phenolic compound content increased after sprouting, reaching 119.94 ± 3.40 mg (100 g)^−1^ in the SO. Regarding avenanthramides, three types were identified in OH (2c, 2p and 2f), while only one in OG (2f), and at a significantly lower concentration; sprouting increased avenanthramide concentration up to 39.2% of the total phenolic compounds found. It has been previously shown that avenanthramides are only found in oats and their concentration increases with sprouting [91,92].

Our results showed that avenanthramide 2c and 2p were identified and quantified in SO but not in OG. This agrees with the fact that a controlled steeping and sprouting process could increase the avenanthramide content in oats [87]. In particular, sprouting improves the degradation of cell wall arabinoxylans by the action of endoxylanases [93], which provides a higher extraction and bioaccessibility of phenolic compounds [94].

In the case of hydrolyzed hull samples, as stated above, EH1-OH and EH2-OH showed a lower diversity of phenolic compounds compared to OH, and the number of phenolic compounds was reduced to 4 and 5, respectively. The enzymatic activity of both enzymes reduced the presence of avenanthramides, regardless of the enzyme used, but increased the solubilization of ferulic acid, p-coumaric, caffeic and 4 hydroxybenzaldehyde. The efficiency of hydrolysis was double in UltraFloXL compared to Viscoferm for EH2-OH) for ferulic acid, p-coumaric acid and caffeic acid. The use of both enzymes increases the solubilization and bioaccessibility of these compounds, from 3 to 30%, when compared to non-hydrolyzed OH.

### 3.3. Total Antioxidant Capacity (TAC)

The antioxidant capacity of the OG, OH, SO and enzymatic hydrolyzed oat hull (EH1-OH and EH2-OH) were assessed through different methodologies: 2,2′-azino-bis (3-ethylbenzothiazoline-6-sulfonic acid) (ABTS^•+^), Ferric reducing ability assay (FRAP) and oxygen radical absorbance capacity (ORAC).

**Figure 3:**
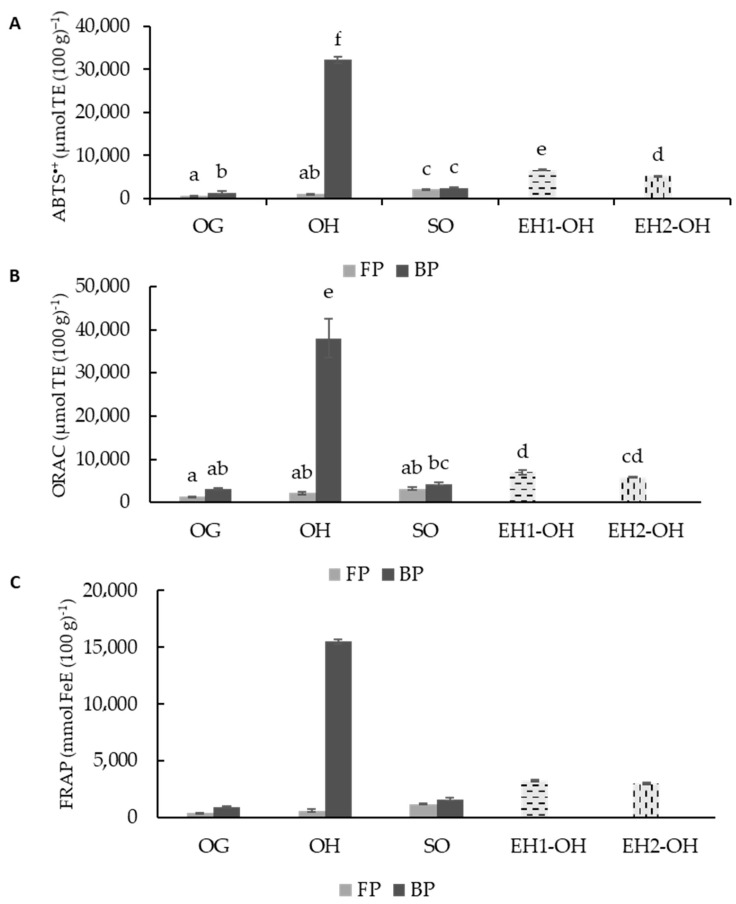
ABTS^•+^, (**A**), ORAC (**B**), FRAP (**C**) values for free phenolic fraction (FP) and bound phenolic fraction (BP) for free polyphenolic fraction (FP) and bound polyphenolic fraction (BP) of oat grain (OG), oat hulls (OH), sprouted oat (SO), enzymatic hydrolysate oat hulls with UltraFloXL (EH1-OH) and enzymatic hydrolysate oat hulls with Viscoferm (EH2-OH) samples. The results were expressed in μmol TE (100 g)^−1^ of dry matter. Different letters indicate significant differences (*p* < 0.05).

ABTS^•+^), was measured in all the samples (Figure 3A); the OH bound fraction showed significantly (*p* < 0.05) higher radical scavenging activity (32,256 µmol TE (100 g)^−1^) than OG (1379 µmol TE (100 g)^−1^) and SO (2068 µmol TE (100 g)^−1^). The sprouting process increased antioxidant activity 1.5 times in reference to the native grain. However, FP fractions behaved differently; no significant differences (*p* ≥ 0.05) were observed between OG (667.74 µmol TE (100 g)^−1^) and OH (1092.35 µmol TE (100 g)^−1^) FP extracts, while SO FP (2068.65 µmol TE (100 g)^−1^) was significantly higher than the other two FP fractions (OG and OH). This increment may respond to the antioxidant contribution of phenolic compounds released during sprouting [95,96]. Avenanthramides, which have a soluble character, may be responsible for the increment observed in the SO-soluble fraction (FP) after sprouting. The mechanism of action of these compounds has been studied, with three main pathways identified: hydrogen atom transfer (HAT), single electron transfer followed by proton transfer and sequential proton loss electron transfer (SPLET) in both polar and non-polar media [97]. The process of enzymatic hydrolysis increased 10 times the ABTS^•+^), radical capacity of the OH FP fraction when using UltrafloxL, and 9.5 times with Viscoferm, probably due to the release of ferulic, *p*-coumaric, caffeic and 4-hydroxybenzaldehyde (Table 2 and Table 3).

After analyzing a range of colored varieties of oat grains and the corresponding hulls, Varga et al. [19] found higher antioxidant activities in OH than in OG. These authors also described bound fractions with higher antioxidant capacities than those of soluble fractions, which is in accordance with the results found in our study. Varga el al. [19] reported a 20-fold increase in phenolic compounds in hull fractions when compared to grain. These differences were not reflected in the antioxidant activity, and the justification for these results was based on the high antioxidant capacity reported for avenanthramides, which is significantly higher than that of the other phenolic compounds present in oat.

A similar trend was observed with the other antioxidant markers analyzed (Figure 3B,C), with some differences. These differences may be explained by the differences between ABTS^•+^), radicals and ORAC. ABTS^•+^), reacts with a higher range of antioxidants, and it is used to determine both hydrophilic and hydrophobic antioxidant activity [98]. It has been observed that most vegetables show much higher antioxidant capacities as measured by ABTS^•^+ assay [99]. Kruma et al. [100] reported that there is a higher diversity of compounds that react to ABTS radicals as compared to DPPH or other antioxidant markers. The ferric reducing antioxidant power (FRAP) assay as an ET-based method measures the reduction of ferric ion (Fe^3+)^ ligand complex to ferrous (Fe^2+^) since it is an important indicator of antioxidant activity in cereals and shows a good correlation with other parameters such as TPs, ABTS^•+^), and ORAC [101]. The values of FRAP were higher in the bound fraction compared to the free fraction regardless of the sample studied and OH showed a better ability to reduce iron, as compared to grain and sprouted grain. The sprouting increases the ability to reduce iron of native grain, as was observed in ABTS^•+^), and ORAC markers. The hydrolysis of OH increases the solubilization of compounds with a high FRAP capacity 5-fold with respect to the OH soluble fraction (Figure 3C).

**Figure 4:**
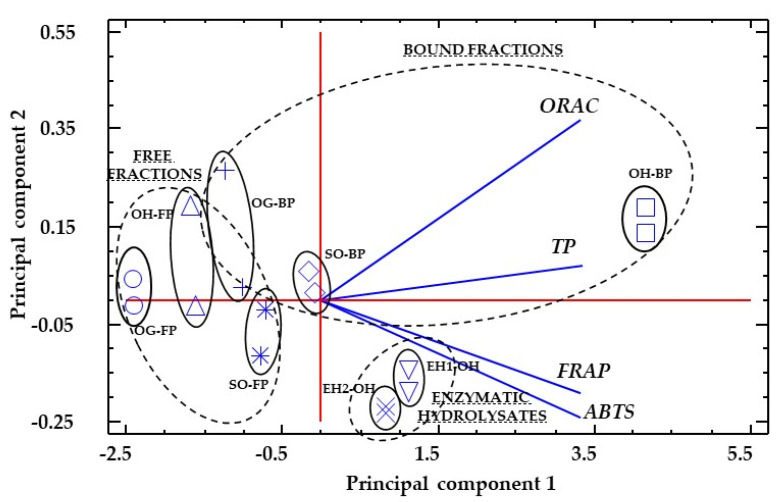
PCA analysis for free phenolic fraction (FP) and bound phenolic fraction (BP) for free polyphenolic fraction (FP) and bound polyphenolic fraction (BP) of oat grain (OG), oat hulls (OH), sprouted oat (SO), enzymatic hydrolysate oat hulls with UltraFloXL (EH1-OH) and enzymatic hydrolysate oat hulls with Viscoferm (EH2-OH) samples.

A principal component analysis (PCA) was carried out using the antioxidant results of the different samples and hydrolysates (Figure 4). The dataset was log-transformed before PCA. The first two principal components (PCs) accounted for 99.2% and 0.6% of the total variation. The PCA analysis separated almost completely soluble from bound fractions, showing that the free fractions had lower antioxidant activities in general, but the higher difference with bound fractions was due to ORAC values. The variability between bound fractions was higher than that found between free fractions, with higher differences due to the TP and ORAC values. Enzymatic hydrolysis resulted in similar antioxidant profiles, with a clearly defined group that showed higher antioxidant (ABTS^•+^) and FRAP) values. The EH1-OH and EH2-OH samples’ different antioxidant profiles to those of the other samples could be based on the higher proportions of caffeic acid when compared to the rest of the phenolics analyzed. Caffeic acid has been reported to have higher antioxidant activity than ferulic and *p*-coumaric acids [102].

### 3.4. Glycemic Index (GI)

**Figure 5:**
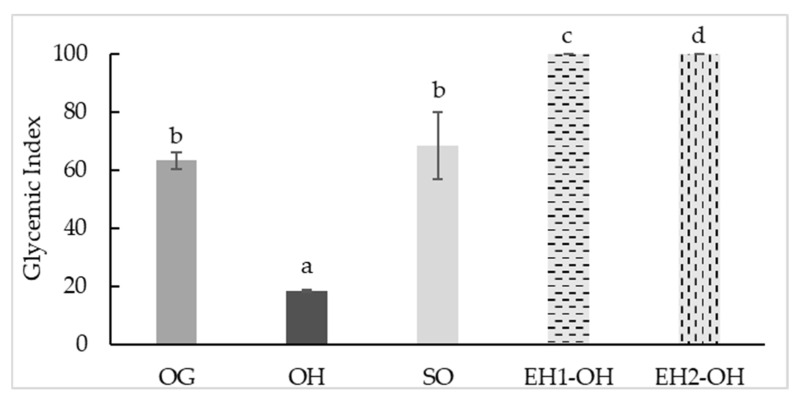
Glycemic Index (GI) values for oat grain (OG), oat hulls (OH), sprouted oat (SO), enzymatic hydrolysate oat hulls with UltraFloXL (EH1-OH) and enzymatic hydrolysate oat hulls with Viscoferm (EH2-OH) samples. Different letters indicate significant differences (*p* < 0.05).

The glycemic index (GI) was evaluated in all samples; the hull showed the lowest GI values (18.47), followed by grain (63.37) and sprouted grain (68.39, Figure 5). The important starch content in oats provides a significant release of glucose in the bloodstream during digestion, but the presence of several bioactive compounds, such as phenolics and β-glucans, can play an important role in glycemia control through different mechanisms [103]. For example, sprouting alters nutrient availability and specifically produces a partial degradation of starch [85], which is reflected in increased GI values; nevertheless, resulting *in vivo* GI is a multifactor outcome, and the increase in bioavailability of phenolics during sprouting can play a role through an inhibitory effect on α-amylase. During oat sprouting, bound phenolics decrease and free and total phenolics increase, especially *p*-coumaric, ferulic acids, and avenanthramides [88]; all of them have been described, especially the last one, along with avenanthramides as potent α-amylase and α glucosidase inhibitors [89].

However, excessive sprouting can lead to the depolymerization of β-glucans due to the action of endogenous β-glucanase [87], which adversely affects the GI of a product, since β-glucans modify the viscosity and GI. Therefore, controlling the degree of sprouting is a potential strategy to optimize oat derivates’ low GI. In addition, the specific and unique lipid composition in oats can be related to a low GI, as a recent study indicated that consuming oat polar lipids could reduce glucose and insulin responses and modulate second meal postprandial metabolic responses [103].

In the present study, no beneficial effects on GI were observed after applying enzymatic hydrolysis with hydrolytic enzymes (UltrafloXl and Viscoferm); indeed, since free monosaccharides and oligosaccharides produced by enzymatic hydrolysis may have the opposite effect, the hydrolysates reached values close to 100 (Figure 5).

**Figure 6:**
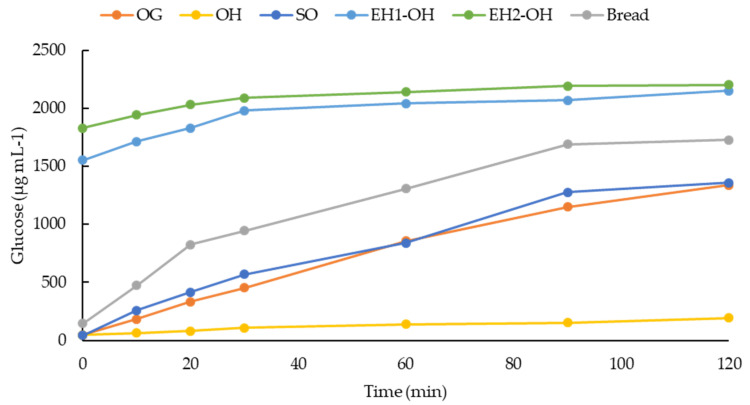
Glucose kinetics consumption (μg mL^−1^) of oat grain (OG), oat hulls (OH), sprouted oat (SO), enzymatic hydrolysate oat hulls with UltraFloXL (EH1-OH) and enzymatic hydrolysate oat hulls with Viscoferm (EH2-OH) samples.

Although GI values are important to determine the health benefits of a product, the rate of conversion into starch is also quite important since foods with the same GI but different rates can produce significant health impacts. As expected, OH showed the lowest rate, followed by OG and OS. The sprouted grain showed a higher rate at the beginning of hydrolysis (40 min). However, the interference of certain types of compounds with α-amylase, as described above, probably reduced the rate, leading to values similar to the native OG. In contrast, hydrolyzed OH showed a high rate, which would translate into a high GI (Figure 6).

### 3.5. Anti-Inflammatory Activity

**Figure 7:**
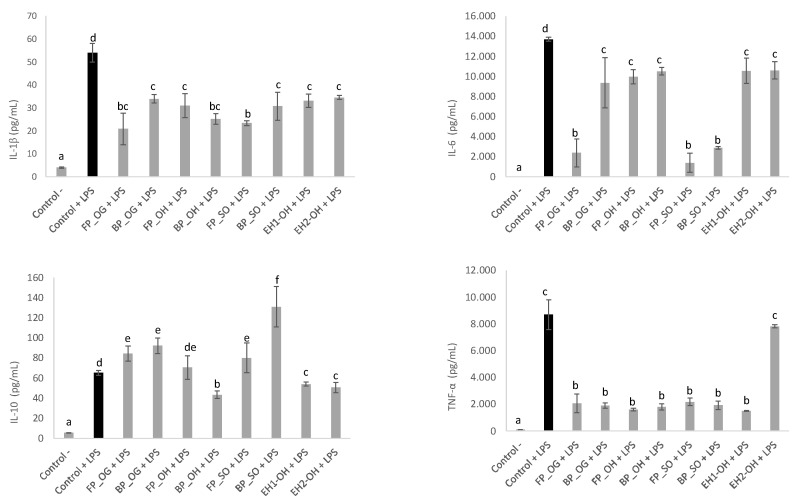
Macrophages cytokine profile of culture supernatants treated with free (FP) and bound (BP) phenolic extracts obtained from OG, OH, SO and EH1-OH and EH2-OH at 0.5 mg/mL. Macrophages treated with growth medium (control-), 100 ng/mL LPS (control+) or 100 ng/mL LPS + free (FP) and bound (BP) phenolic fractions for 24 h. Data are means ± standard deviations (*n* = 4). Different letters denote statistical differences among the experimental groups. Abbreviations: LPS: lipolysaccharide from *Escherichia coli*; OG: oat grain; OH: oat hull; SO: sprouted oat; EH1-OH: enzymatic hydrolysate oat hulls with UltraFlo XL; EH2-OH: enzymatic hydrolysate oat hulls with Viscoferm.

To determine whether FP and BP extracts from oat samples displayed modulatory effects, the protein levels of several immune mediators were measured in murine macrophages in the presence of a pro-inflammatory insult, such as the Gram-negative bacteria endotoxin LPS (Figure 7). As compared with non-stimulated cells (control -), LPS (control +) significantly induced the secretion of cytokines IL-1β, IL-6, IL-10 and TNF-α (*p* < 0.05). Overall, the overproduction of pro-inflammatory cytokines was significantly reverted in the presence of all the oat phenolic extracts at 0.5 mg/mL (*p* < 0.05). Moreover, FP and BP from OG and SO increased the levels of IL-10 (*p* < 0.05)—an anti-inflammatory cytokine able to inhibit the synthesis of proinflammatory cytokines in macrophages. Our results agree with existing evidence supporting the anti-inflammatory properties of oats attributed mainly to avenanthramides and β-glucans [104].

The wide variation in the diversity and abundance of phenolic compounds among oat samples was not associated with a high variation in the levels of the cytokines studied. The most outstanding differences were observed for the anti-inflammatory effects exerted by OG (FP) and SO (either FP or BP) extracts, showing stronger inhibition of IL-1β and IL-6 secretion and higher production of the anti-inflammatory cytokine IL-10. OG phenolic fraction was characterized by poor diversity and low TP content (including avenanthramides) as compared to the other studied samples; therefore, the highest β-glucan content present in OG FP extracts could be a plausible explanation of our results. In the case of SO, observed findings could be attributed to the specific higher abundance of avenanthramides in the FP fraction, the high diversity of phenolic acids and the presence of avenanthramides in the BP. In particular, oat avenanthramides exert anti-inflammatory effects through inhibition of NF-kB-mediated inflammatory response, as reported in *in vitro* research studies using different cell lines [105].

## 4. Conclusions

Sprouting produced significant increases of free and bound phenols with respect to native oat grain, with special impact on ferulic acid, caffeic acid and avenanthramide isoforms (2c, 2p and 2f), enhancing the antioxidant and anti-inflammatory properties of the whole oat grain. Additionally, the process favored the reduction of antinutrients such as phytic acid, which is a potent inhibitor of iron, zinc and calcium absorption.

On the other hand, the hydrolysis of the hull increased 4.5- and 5-fold the release of bound phenols using Viscoferm (EH2-OH) and Ultraflo XL (EH21-OH), respectively, compared to the non-hydrolyzed hull, releasing especially ferulic acid, which increased 6-fold and *p*-coumaric (11-fold increase), as compared to the content in grain. In addition, Viscoferm increased the solubilization of β-glucan up to levels of those of native grains.

Moreover, the use of Viscoferm increased 4.55-fold times soluble β-glucans in the OH, reaching the hull hydrolyzed values similar to the oat grain (4.04 vs. 4.46 g (100 g)^−1^. Since healthy benefits are based on a nutritional and bioactive balance, the use of combined germinated and hydrolytic ingredients can provide important benefits, reducing the drawbacks of the independent processes, such as reduction of β-glucan in germination or increment of GI after the hydrolytic process.

This study shows strategies (germination and hydrolysis) that can provide interesting opportunities for the development of functional ingredients, reducing the amount of byproducts (hull) and enhancing the properties of ingredients produced from sprouted whole grain.

Additionally, the use of both combined strategies can help to reduce the volume of byproducts produced, favoring the implementation of sustainable circular strategies in the food industry.

## Figures and Tables

**Table 1:** Nutritional characterization of oat grain (OG), oat hulls (OH) and sprouted oat (SO) samples. Values were expressed as g (100 g)^−1^ of dry matter and fatty acids as % over total fatty acid content.

	OG	OH	SO
**Ash**	2.5 ± 0.09 ^a^	4.3 ± 0.00 ^b^	2.41 ± 0.20 ^a^
**Carbohydrates**	79.07 ± 0.12 ^b^	92.04 ± 0.07 ^c^	76.62 ± 0.89 ^a^
**TDF**	12.63 ± 0.13 ^b^	89.64 ± 0.10 ^c^	8.81 ± 0.77 ^a^
**β** **-glucan**	4.46 ± 0.16 ^c^	0.11 ± 0.06 ^a^	1.55 ± 0.04 ^b^
**TSC**	55.07 ± 1.10 ^b^	2.89 ± 0.48 ^a^	59.01 ± 2.70 ^c^
**Fat**	6.73 ± 0.08 ^b^	0.61 ± 0.01 ^a^	9.45 ± 0.75 ^c^
SFA	18.00 ± 0.00 ^a^	34.83 ± 1.65 ^c^	20.41 ± 0.00 ^b^
MUFA	37.00 ± 0.00 ^a^	40.30 ± 0.42 ^b^	42.86 ± 0.00 ^c^
PUFA	45.00 ± 0.00 ^c^	24.87 ± 1,23 ^a^	36.73 ± 0.00 ^b^
Palmitic acid (C16:0)	15.00 ± 0.00 ^a^	34.83 ± 1.65 ^c^	16.33 ± 0.00 ^b^
Stearic acid (C18:0)	n.d.	n.d.	2.04 ± 0.00 ^a^
Oleic acid (C18:1)	37.00 ± 0.00 ^a^	40.30 ± 0.42 ^b^	42.86 ± 0.00 ^c^
Linoleic acid (C18:2)	45.00 ± 0.00 ^c^	24.87 ± 1.23 ^a^	36.73 ± 0.00 ^b^
Behenic acid (C122:0)	n.d.	n.d.	2.04 ± 0.00 ^a^
C18:1/C18:2	0.82 ± 0.00 ^a^	1.62 ± 0,06 ^c^	1.17 ± 0.00 ^b^
**Protein**	11.69 ± 0.04 ^c^	3.06 ± 0.07 ^a^	11.52 ± 0.06 ^b^
**Moisture**	10.16 ± 0.01 ^c^	8.03 ± 0.02 ^b^	6.93 ± 0.01 ^a^
**PA**	0.94 ± 0.01 ^c^	0.07 ± 0.00 ^a^	0.83 ± 0.01 ^b^

Abbreviations: TDF: total dietary fiber, TSC: total starch content, SFA: saturated fatty acid, MUFA: monounsaturated fatty acid, PUFA: polyunsaturated fatty acid, C18:1/C18:2: oleic acid/linoleic acid, PA: phytic acid. n.d.: not detected. Different letters in the same row indicate significant differences (*p* < 0.05).

**Table 2:** Phenolic compounds identified in oat samples by HPLC-ESI-QTOF-MS.

Class	Sub-class	Compound	Formula	Fragments	OG		OH		SO		EH1-OH	EH2-OH
					Error	Fraction	Error	Fraction	Error	Fraction	Error	Error
**Phenolic acids**	**Hydroxybenzoic acids**	**Protocatechuic acid**	C_7_H_6_O_4_	151, 136, 112	-4.34	FP	n.d.	n.d.	3.46	BP	n.d.	n.d.
		Hydroxybenzoic acid	C_7_H_6_O_3_	135, 125, 121	-3.49	BP	-2.05	FP	4.47	BP	n.d.	3.75
	Hydroxycinnamic acids	Ferulic acid	C_10_H_10_O_4_	178, 149, 134	-1.89	BP	57.88	BP	0.17	BP	2.23	1.2
		*p*-Coumaric acid	C_9_H_8_O_3_	119	n.d.	n.d.	-2.64	BP	n.d.	n.d.	1.63	-2.03
		Sinapic acid	C_11_H_12_O_5_	208	-2.24	BP	n.d.	n.d.	-0.91	BP	n.d.	n.d.
		Avenantrahamide 2c	C_16_H_13_NO_6_	270, 178, 161	n.d.	n.d.	-0.28	FP	0.99	FP-BP	n.d.	n.d.
		Avenanthramide 2p	C_16_H_13_NO_5_	254, (226), (160)	n.d.	n.d.	-4.03	FP	9.01	FP	n.d.	n.d.
		Avenanthramide 2f	C_17_H_15_NO_6_	284	-2.25	FP	-2.55	FP	1.4	FP-BP	n.d.	n.d.
		Diferulic isomer 1	C_20_H_18_O_8_	341, (282), 193, (112)	-1.58	BP	n.d.	n.d.	1.79	BP	n.d.	n.d.
		Diferulic isomer 2	C_20_H_18_O_8_	359, 341, 326	-3.65	BP	n.d.	n.d.	-1.58	BP	n.d.	n.d.
		Diferulic isomer 4	C_20_H_18_O_8_	341, 326, 282, 248, 227	-3.39	BP	n.d.	n.d.	-2.35	BP	n.d.	n.d.
		Diferulic isomer 5	C_20_H_18_O_8_	347, 313, 261, 217, 193, 178	-4.69	BP	n.d.	n.d.	-0.54	BP	n.d.	n.d.
		Diferulic isomer 6	C_20_H_18_O_8_	341, 303, 239, 193, 178	-0.54	BP	n.d.	n.d.	n.d.	n.d.	n.d.	n.d.
		Caffeic acid	C_9_H_8_O_4_	(165), 135, 127	-5.1	FP-BP	7.68	BP	-1.76	BP	-2.32	-5.1
		Isoferulic acid	C_10_H_10_O_4_	178, 149, 134	-1.38	BP	n.d.	n.d.	-0.35	BP	n.d.	n.d.
		1-O-Sinapoyl-beta-D-glucose	C_17_H_22_O_10_	216, 162, 119	-0.98	FP	n.d.	n.d.	-0.46	FP	n.d.	n.d.
Flavonoids	Flavones	Apigenin-6-C-arabinoside-8-C-hexoside III	C_26_H_28_O_14_	221, 137	n.d.	n.d.	-1.19	FP	0.05	FP	n.d.	n.d.
Others	Hydroxybenzaldehyde acids	4-Hydroxybenzaldehyde	C_7_H_6_O_2_	(112)	0.84	BP	-3.25	FP-BP	0.84	BP	1.66	-8.99

Abbreviations: OG: oat grain, OH: oat hulls, SO: sprouted oat, EH1-OH: enzymatic hydrolysate oat hulls with UltraFloXL, EH2-OH: enzymatic hydrolysate oat hulls with Viscoferm, FP: free polyphenols fraction, BP: bound polyphenols fraction, n.d.: not detected. Fragments in parentheses are minority. Error expressed in ppm.

**Table 3:** Phenolic compounds (mg (100 g)^−1^ of dry matter) quantified in oat samples by HPLC-ESI-QTOF-MS.

Class	Sub-class	Compound	OG		OH		SO		EH1-OH	EH2-OH
			FP	BP	FP	BP	FP	BP		
**Phenolic acids**	**Hydroxybenzoic acids**	Protocatechuic acid	1.05 ± 0.04 ^a^	n.d.	n.d.	n.d.	n.d.	1.71 ± 0.17 ^b^	n.d.	n.d.
		Hydroxybenzoic acid	n.d.	0.75 ± 0.01 ^c^	0.53 ± 0.02 ^a^	n.d.	n.d.	0.66 ± 0.10 ^b^	n.d.	0.74 ^c^
	Hydroxycinnamic acids	Ferulic acid	0.36 + 0.01 ^a^	28.30 ± 1.56 ^d^	0.50 + 0.13 ^a^	213.76 ± 4.39 ^f^	n.d.	32.36 ± 3.09 ^e^	6.15 ± 0.87 ^c^	3.38 ^b^
		p-Coumaric acid	n.d.	n.d.	n.d.	130.67 ± 1.43 ^c^	n.d.	n.d.	11.47 ± 2.11 ^b^	7.32 ^a^
		Sinapic acid	n.d.	2.93 ± 0.18 ^b^	n.d.	n.d.	n.d.	2.50 ± 0.14 ^a^	n.d.	n.d.
		Avenanthramide 2c	n.d.	n.d.	0.21 ± 0.02 ^a^	n.d.	13.40 ± 0.34 ^b^	0.19 ± 0.04 ^a^	n.d.	n.d.
		Avenanthramide 2p	n.d.	n.d.	1.58 ± 0.14 ^a^	n.d.	14.35 ± 0.27 ^b^	n.d.	n.d.	n.d.
		Avenanthramide 2f	0.23 ± 0.07 ^a^	n.d.	1.48 ± 0.10 ^b^	n.d.	19.24 ± 0.19 ^d^	1.67 ± 0.01 ^c^	n.d.	n.d.
		Diferulic isomer 1	n.d.	4.42 ± 0.22 ^b^	n.d.	n.d.	n.d.	3.80 ± 0.38 ^a^	n.d.	n.d.
		Diferulic isomer 2	n.d.	5.02 ± 0.63 ^b^	n.d.	n.d.	n.d.	3.85 ± 0.38 ^a^	n.d.	n.d.
		Diferulic isomer 4	n.d.	5.70 ± 0.39 ^a^	n.d.	n.d.	n.d.	8.35 ± 0.82 ^b^	n.d.	n.d.
		Diferulic isomer 5	n.d.	6.83 ± 0.59 ^a^	n.d.	n.d.	n.d.	7.30 ± 0.62 ^a^	n.d.	n.d.
		Diferulic isomer 6	n.d.	0.74 ± 0.06 ^a^	n.d.	n.d.	n.d.	n.d.	n.d.	n.d.
		Caffeic acid	2.32 ± 0.01 ^c^	1.50 ± 0.11 ^a^	n.d.	6.42 ± 0.26 ^e^	n.d.	3.44 ± 0.41 ^d^	2.41 ± 0.25 ^c^	1.72 ^b^
		Isoferulic acid	n.d.	3.36 ± 0.67 ^a^	n.d.	n.d.	n.d.	2.96 ± 0.39 ^a^	n.d.	n.d.
		1-O-Sinapoyl-beta-D-glucose	1.55 ± 0.02 ^a^	n.d.	n.d.	n.d.	2.45 ± 0.02 ^b^	n.d.	n.d.	n.d.
Flavonoids	Flavones	Apigenin-6-C-arabinoside-8-C-hexoside III	n.d.	n.d.	< LOD	n.d.	< LOD	n.d.	n.d.	n.d.
Others	Hydroxybenzaldehide acids	4-Hydroxybenzaldehyde	n.d.	1.10 ± 0.11 ^a^	4.17 ± 0.06 ^c^	27.88 ± 1.44 ^e^	n.d.	1.71 ± 0.11 ^b^	9.10 ± 1.05 ^d^	9.91 ^d^

Abbreviations: OG: oat grain, OH: oat hulls, SO: sprouted oat, EH1-OH: enzymatic hydrolysate oat hulls with UltraFloXL, EH2-OH: enzymatic hydrolysate oat hulls with Viscoferm, FP: free polyphenols fraction, BP: bound polyphenols fraction, n.d.: not detected. LOD: limit of detection. Different letters indicate significant differences (*p* < 0.05).

## Data Availability

Data are not available.

## Supplementary Materials

The following supporting information can be downloaded at: https://www.mdpi.com/article/10.3390/foods11182769/s1, Table S1. m/z values of phenolic compounds obtained in oat samples by HPLC-ESI-QTOF-MS. Abbreviations: OG: oat grain, OH: oat hulls, SO: sprouted oat, EH1- OH: enzymatic hydrolysate oat hulls with UltraFloXL, EH2-OH: enzymatic hydrolysate oat hulls with Viscoferm, n.d.: not detected; Figure S1. β-glucan values of enzymatic hydrolysate oat hull with UltraFloXL (EH1-OH) and enzymatic hydrolysate oat hull with Viscoferm (EH2-OH) samples. Different letters indicate significant differences (*p* < 0.05); Figure S2. Phytic acid values of enzymatic hydrolysate oat hull with UltraFloXL (EH1-OH) and enzymatic hydrolysate oat hull with Viscoferm (EH2-OH) samples. Different letters indicate significant differences (*p* < 0.05).
