# Supplementary material for: Sprouting and Hydrolysis as Biotechnological Tools for Development of Nutraceutical Ingredients from Oat Grain and Hull
Source: Foods. 2022 Sep 8;11(18):2769. doi: 10.3390/foods11182769 (PMC9497932; doi:10.3390/foods11182769)
Supplement: Supplementary file 1 [file foods-11-02769-s001.zip › foods-1854377-supplementary.pdf]

Supplementary Materials:

Table S1. m/z values of phenolic compounds obtained in oat samples by HPLC-ESI-QTOF-MS. Abbreviations: OG: oat grain, OH: oat hulls, SO: sprouted oat, EH1-OH: enzymatic hydrolysate oat hulls with UltraFloXL, EH2-OH: enzymatic hydrolysate oat hulls with Viscoferm, n.d.: not detected.

Compound	OG		OH		SO		EH1-OH		EH2-OH	
	m/z exp	*m/z* calc	*m/z* exp	*m/z* calc	*m/z* exp	*m/z* calc	*m/z* exp	*m/z* calc	*m/z* exp	*m/z* calc
Protocatechuic acid		153.0193	n.d.	n.d.	153.0188	153.0193	n.d.	n.d.	n.d.	n.d.
Hydroxybenzoic acid	137.0249	137.0244	137.0247	137.0244	137.0238	137.0244	n.d.	n.d.	137.0239	137.0244
Ferulic acid	193.051	193.0506	193,0394	193,0506	193.0506	193.0506	193.0502	193.0506	193.0504	193.0506
p-Coumaric acid	n.d.	n.d.	163.0405	163.0401	n.d.	n.d.	163.0398	163.0401	163.0404	163.0401
Sinapic acid	223.0617	223.0612	n.d.	n.d.	223.0614	223.0612	n.d.	n.d.	n.d.	n.d.
Avenantrahamide C	n.d.	n.d.	314.0671	314.0670	314.0667	314.0670	n.d.	n.d.	n.d.	n.d.
Avenanthramide 2p	n.d.	n.d.	298.0733	298.0721	298.0694	298.0721	n.d.	n.d.	n.d.	n.d.
Avenanthramide 2f	328.0834	328.0827	328.0835	328.0827	328.0822	328.0827	n.d.	n.d.	n.d.	n.d.
Diferulic isomer 1	385.0935	385.0929	n.d.	n.d.	385.0922	385.0929	n.d.	n.d.	n.d.	n.d.
Diferulic isomer 2	385.0943	385.0929	n.d.	n.d.	385.0935	385.0929	n.d.	n.d.	n.d.	n.d.
Diferulic isomer 4	385.0942	385.0929	n.d.	n.d.	385.0938	385.0929	n.d.	n.d.	n.d.	n.d.
Diferulic isomer 5	385.0947	385.0929	n.d.	n.d.	385.0931	385.0929	n.d.	n.d.	n.d.	n.d.
Diferulic isomer 6	385.0931	385.0929	n.d.	n.d.	n.d.	n.d.	n.d.	n.d.	n.d.	n.d.
Caffeic acid	179.0359	179.0350	179.0336	179.0350	179.0353	179.0350	179.0354	179.0350	179.0359	179.0350
Isoferulic acid	193.0509	193.0506	n.d.	n.d.	193.0507	193.0506	n.d.	n.d.	n.d.	n.d.
1-O-Sinapoyl-beta-D-glucose	385.1144	385.1140	n.d.	n.d.	385.1142	385.114	n.d.	n.d.	n.d.	n.d.
Apigenin-6-C-arabinoside-8-C-hexoside III	n.d.	n.d.	563.1413	563.1406	563.1406	563.1406	n.d.	n.d.	n.d.	n.d.
4-Hydroxybenzaldehyde	121.0294	121.0295	121.0299	121.0295	121.0294	121.0295	121.0293	121.0295	121.0306	121.0295

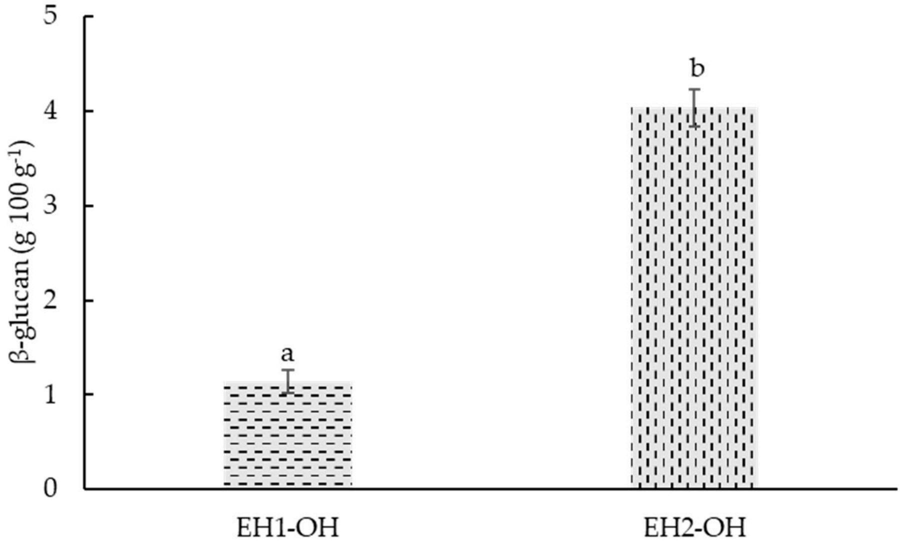

Figure S1. β-glucan values of enzymatic hydrolysate oat hull with UltraFloXL (EH1-OH) and enzymatic hydrolysate oat hull with Viscoferm (EH2-OH) samples. Different letters indicate significant differences ($p < 0.05$).

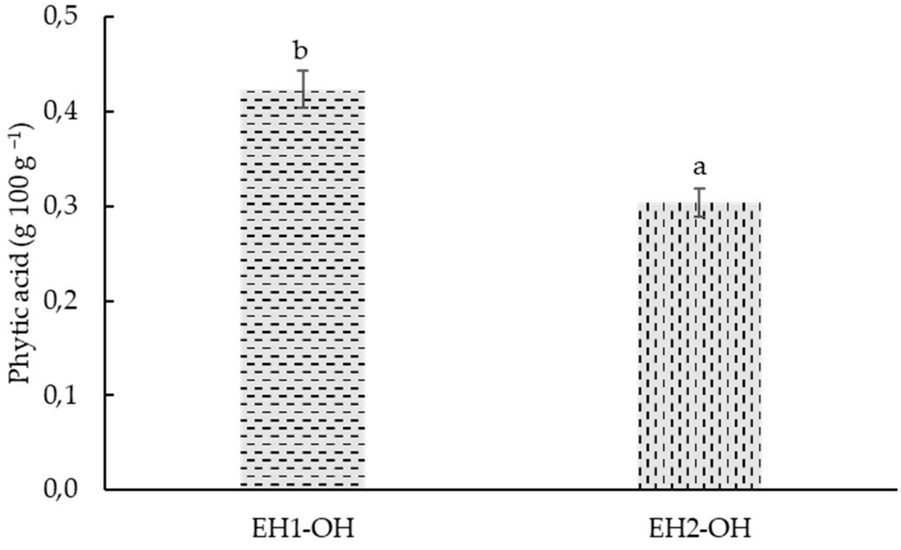

Figure S2. Phytic acid values of enzymatic hydrolysate oat hull with UltraFloXL (EH1-OH) and enzymatic hydrolysate oat hull with Viscoferm (EH2-OH) samples. Different letters indicate significant differences ($p < 0.05$).
